# Hepatoid Adenocarcinoma of the Stomach Combined With Gastric Neuroendocrine Carcinoma: A Case Report

**DOI:** 10.1002/ccr3.70966

**Published:** 2025-10-31

**Authors:** Banghui Ma, Ping Zheng, Yongdong Jin

**Affiliations:** ^1^ Department of Medical Oncology, Sichuan Clinical Research Center for Cancer, Sichuan Cancer Hospital & Institute, Sichuan Cancer Center, School of Medicine University of Electronic Science and Technology of China Chengdu China; ^2^ Department of Pathology, Sichuan Clinical Research Center for Cancer, Sichuan Cancer Hospital & Institute, Sichuan Cancer Center University of Electronic Science and Technology of China Chengdu China; ^3^ Department of Medical Oncology, Sichuan Clinical Research Center for Cancer, Sichuan Cancer Hospital & Institute, Sichuan Cancer Center University of Electronic Science and Technology of China Chengdu China

**Keywords:** case report, gastric neuroendocrine carcinoma, hepatoid adenocarcinoma of the stomach, immunotherapy, liver metastasis, targeted therapy

## Abstract

Both hepatoid adenocarcinoma of the stomach (HAS) and gastric neuroendocrine carcinoma (NEC) are rare subtypes of gastric cancer, characterized by aggressive behavior and poor prognosis, for which no definitive treatment regimen has been established to date. A 56‐year‐old male was diagnosed with HAS combined with NEC, accompanied by perigastric lymph node and liver metastases. Initially, he received EP chemotherapy, which was subsequently switched to a regimen of capecitabine plus oxaliplatin due to insufficient therapeutic response. Ultimately, the treatment was transitioned to a combination of immunotherapy and targeted therapy with Lenvatinib plus Envafolimab, owing to the development of capecitabine intolerance. The optimal management of this disease remains undefined, and conventional chemotherapy often results in suboptimal outcomes. However, in this case, the treatment with Lenvatinib plus Envafolimab appears to be an effective strategy for prolonging survival time and improving quality of life. Further experimental and clinical investigations are warranted to validate these findings and substantiate this therapeutic hypothesis.

## Graphical abstract

(a) Gastric resection (52‐year‐old man, initial presentation): poorly differentiated carcinoma, consistent with hepatoid adenocarcinoma with neuroendocrine differentiation. Carcinoma invaded the muscularis propria, and vascular tumor emboli were identified. (b, c) Hepatic resection specimen: poorly differentiated carcinoma was detected, representing metastatic hepatoid adenocarcinoma. A small number of cancer cells were found with perineural invasion, focal perineural invasion correlates with the patient's rapid local recurrence and underscores the rationale for adding lenvatinib/envafolimab after failure of cisplatin‐based and capecitabine‐containing regimens.

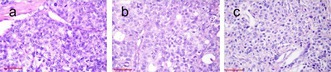

**Summary**

- Hepatoid adenocarcinoma combined with gastric neuroendocrine carcinoma is rare and aggressive.
- In this case, Lenvatinib plus Envafolimab showed clinical benefit after failure of standard chemotherapy, suggesting this combination may be a viable option for patients with advanced disease.

## 1 Introduction

Gastric neuroendocrine carcinoma (NEC) represents a rare subtype of gastric cancer, accounting for < 1% of all diagnosed cases. This subtype is characterized by a high metastatic potential and is associated with a poor prognosis [1]. Currently, no standardized treatment regimen has been established for NEC, as the majority of available evidence is derived from individual case reports. Hepatoid adenocarcinoma of the stomach (HAS) is another uncommon subtype of gastric cancer, histologically resembling hepatocellular carcinoma, and marked by a low incidence and a poor 1‐year survival rate [2]. In 1975, McIntire et al. first reported a gastrointestinal malignancy capable of secreting alpha‐fetoprotein (AFP) [3], and in 1986, Ishikura et al. documented seven cases of gastric adenocarcinoma with elevated serum AFP, including the first case of HAS [4]. Although HAS and NEC are individually rare entities, their co‐occurrence within a single tumor is exceedingly uncommon, with only a limited number of cases reported in both domestic and international literature. In 2012, Akira Suzuki et al. described a case of a composite gastric tumor composed of AFP‐producing carcinoma/hepatoid adenocarcinoma, NEC, and tubular adenocarcinoma [5]. In 2015, Andrzej et al. documented a rare instance of α‐Fetoprotein‐Producing Hepatoid Gastric Adenocarcinoma exhibiting neuroendocrine differentiation [6]. For patients diagnosed with gastric hepatoid adenocarcinoma or neuroendocrine carcinoma, treatment typically involves surgical intervention supplemented by conventional chemotherapeutic agents such as oxaliplatin, capecitabine, and paclitaxel, which have shown limited efficacy in patients with advanced HAS or NEC. In such cases, patients often experience disease progression shortly after receiving standard treatment. Okamoto et al. reported a composite gastric tumor composed of AFP and NEC, and the patient died of liver metastasis 4 years after surgical treatment [7]. Nunobe S et al. reported a compound tumor composed of poorly differentiated adenocarcinoma and simultaneous expression of NEC. The patient died of lymph node metastasis and peritoneal dissemination 16 months after surgical treatment and TS‐1 chemotherapy [8].

However, there is a paucity of evidence‐based guidelines for the effective treatment of patients presenting with concurrent hepatoid adenocarcinoma and neuroendocrine carcinoma of the stomach. In this report, we present a patient diagnosed with HAS and NEC. Following initial surgical resection and adjuvant chemotherapy, the treatment efficacy was suboptimal, resulting in multiple metastases to the liver and other sites. Subsequently, the patient received partial hepatectomy and microwave ablation. For the past 2 years, the patient has been undergoing a regimen of immunotherapy in combination with targeted therapy. Given the absence of therapeutic options underpinned by robust scientific data for this rare disease, exploring various treatment modalities is warranted. Immunotherapy combined with targeted therapy represents a promising avenue for future investigation.

## 2 Case History

**FIGURE 1:**
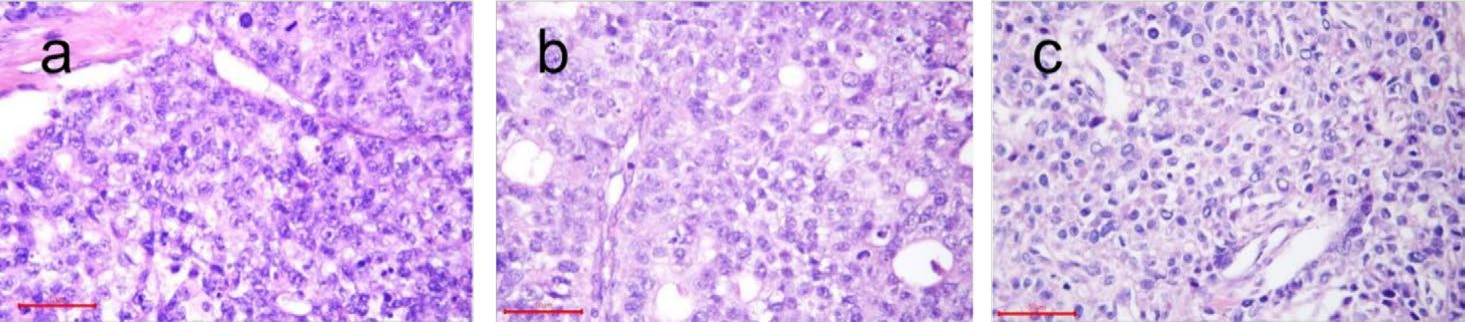
Gastric resection specimen: poorly differentiated carcinoma, consistent with hepatoid adenocarcinoma with neuroendocrine differentiation. Carcinoma invaded the muscularis propria, and vascular tumor emboli were identified (a). Hepatic resection specimen: poorly differentiated carcinoma was detected, representing metastatic hepatoid adenocarcinoma. A small number of cancer cells were found with perineural invasion (b, c).

A 56‐year‐old male patient is currently receiving immunotherapy in combination with targeted therapy following gastric cancer surgery and the presence of multiple abdominal metastases. He was initially diagnosed with gastric cancer 4 years prior, presented with recurrent epigastric pain. The patient has no significant family history of medical illness and no history of smoking or alcohol consumption. Upon examination, vital signs were stable, and the physical examination was largely unremarkable, except for the presence of a surgical scar on the abdomen. No palpable abdominal masses were detected, and there was no tenderness or rebound tenderness observed. Subsequently, the patient underwent laparoscopic exploration, minimally invasive distal gastrectomy, and adjuvant treatment, including regional arterial perfusion and extended‐release chemotherapy, for abdominal malignant neoplasm. Postoperative pathological analysis confirmed the presence of gastric cancer, with findings indicating a localized adenoid structure (Figure 1). Additionally, four groups of lymph node metastases were identified. Immunohistochemical (IHC) analysis revealed HER2 (0), MLH1(+), MSH2(+), MSH6(+), PMS2(+), PD‐1(+), while other markers indicated CgA(+), Syn(+), histologically characterizing the tumor as neuroendocrine carcinoma with a MIB‐1 index of 60% in tumor cells. Genome sequencing demonstrated microsatellite stability (MSS) in the tumor tissues. The tumor exhibited a combined positive score (CPS) of < 1 and a tumor proportion score (TPS) of < 1, indicating negative PD‐L1 expression.

**FIGURE 2:**
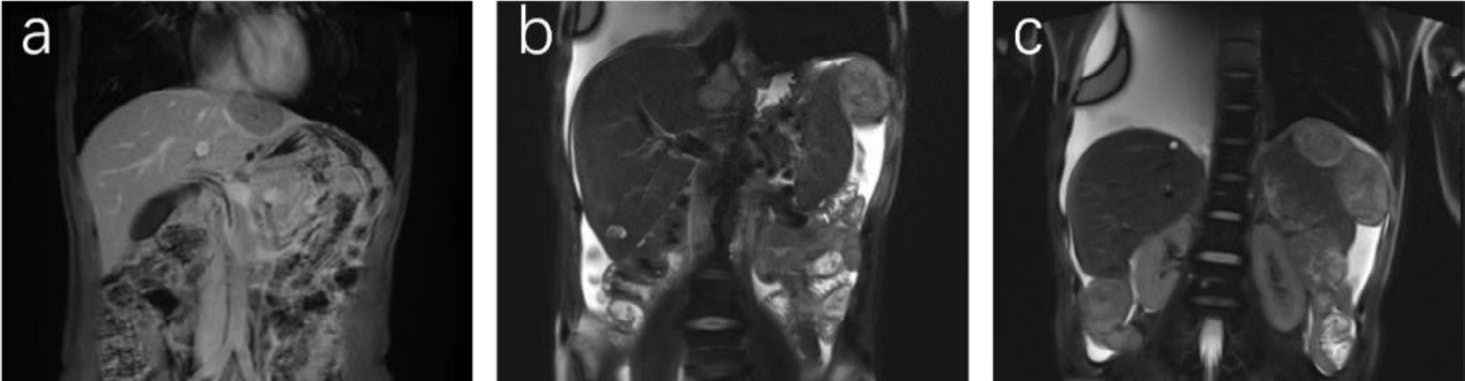
Tumor liver metastasis in 2022. MR in January 2022: Abnormal signal lump in the superior segment of the left lateral lobe of the liver, potentially representing a new liver metastasis (a). MR in August 2022: Multiple nodules, masses located in the left hepatic lobe adjacent to the incisal margin and inferior segment of the right posterior lobe, potentially representing new liver metastases (b, c).

The patient underwent six cycles of the EP chemotherapy regimen, consisting of VP16 100 mg d1‐5 plus DDP 40 mg d1‐3 q3w, starting in January 2021. In January 2022, 8 months post‐chemotherapy, magnetic resonance imaging (MRI) revealed a mass in the superior segment of the left lateral lobe of the liver, suspected to be a metastasis (Figure 2). Subsequently, the patient received two additional cycles of an alternative EP chemotherapy regimen, but the evaluation indicated progressive disease (PD). In April 2022, the patient underwent laparoscopic resection of a complex hepatocellular carcinoma in the left liver, intestinal adhesion release, and gastropexy. Postoperative pathological analysis, interpreted in the context of the patient's clinical history, was consistent with metastatic poorly differentiated carcinoma (Figure 1). Genome sequencing showed PD‐L1:CPS:3, TP53 mutation (54% abundance), PTPRT mutation (32% abundance). IHC showed HepPar‐1(+), AFP(+), Arg‐1(−), GS(+), GPC3(+), CD34(−), VILLIN(+), CgA(+), Syn(+), Ki‐67 (MIB‐1) (+80%), indicating NEC (large cell type) and HAS with bidirectional differentiation. Considering the clinical manifestations, physical signs, pathological results, and imaging findings, other conditions such as hepatic carcinoma and conventional gastric cancer were excluded.

The patient's disease stage, metastatic status, serum AFP levels, and various treatment options will influence survival and prognosis. Most patients with HAS are diagnosed at stage III or IV, often presenting with lymph node and liver metastases. Various studies indicate that the 5‐year survival rate ranges between 8.3% and 34.0% [9, 10, 11].

Following surgery, the patient received one cycle of chemotherapy (oxaliplatin 200 mg plus capecitabine 1500 mg po bid d1‐d14 Q3W). However, the treatment was discontinued due to the development of fatigue and weakness associated with the administration of oral capecitabine. A follow‐up MRI conducted 4 months post‐surgery revealed multiple soft tissue nodules and masses located along the incisal margin of the left hepatic lobe, the hepatosplenic peripheral area, and the pelvic floor peritoneal area, which were suspected to be metastases (Figure 2). Consequently, we initiated a treatment regimen combining immunotherapy with targeted therapy (Lenvatinib capsules 8 mg po qd plus Envafolimab 150 mg ih QW).

**FIGURE 3:**
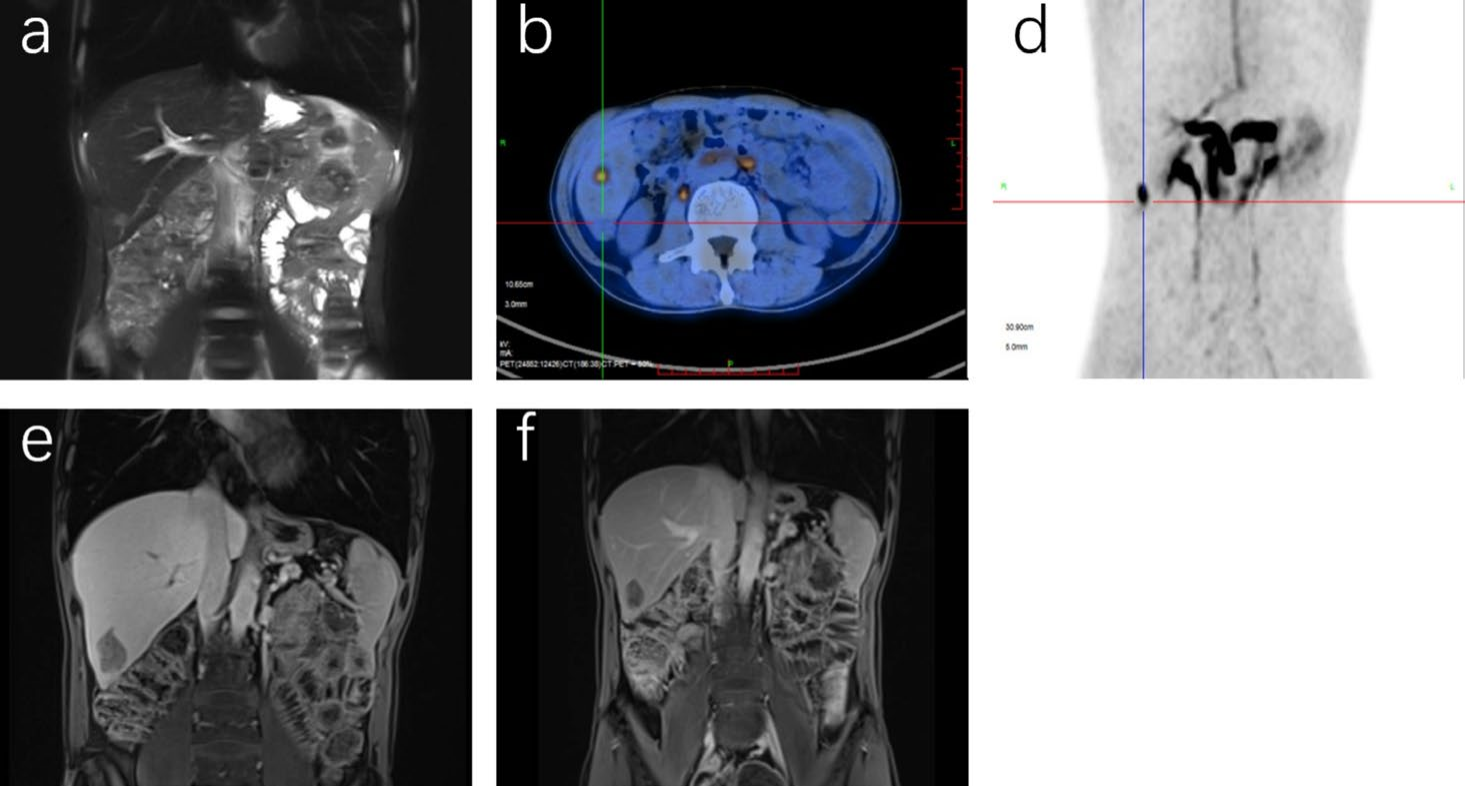
Tumor liver metastasis in 2024. MRI in April 2024 showed a nodule in the inferior segment of the right posterior hepatic lobe, which may represent an additional liver metastasis (a). PET/CT in April 2024 showed a nodule in the right posterior lobe of the liver with increased metabolism, suggesting a new metastasis (b, c). MRI in July 2024 showed that the boundary of the lower segment of the right posterior lobe of the liver was enlarged, which was considered to be a change after the ablation of liver metastases (d). MRI reexamination in September 2024 showed that the nodules in the inferior segment of the right posterior lobe had slightly decreased in size compared to those observed in July 2024 (e, f).

**FIGURE 4:**
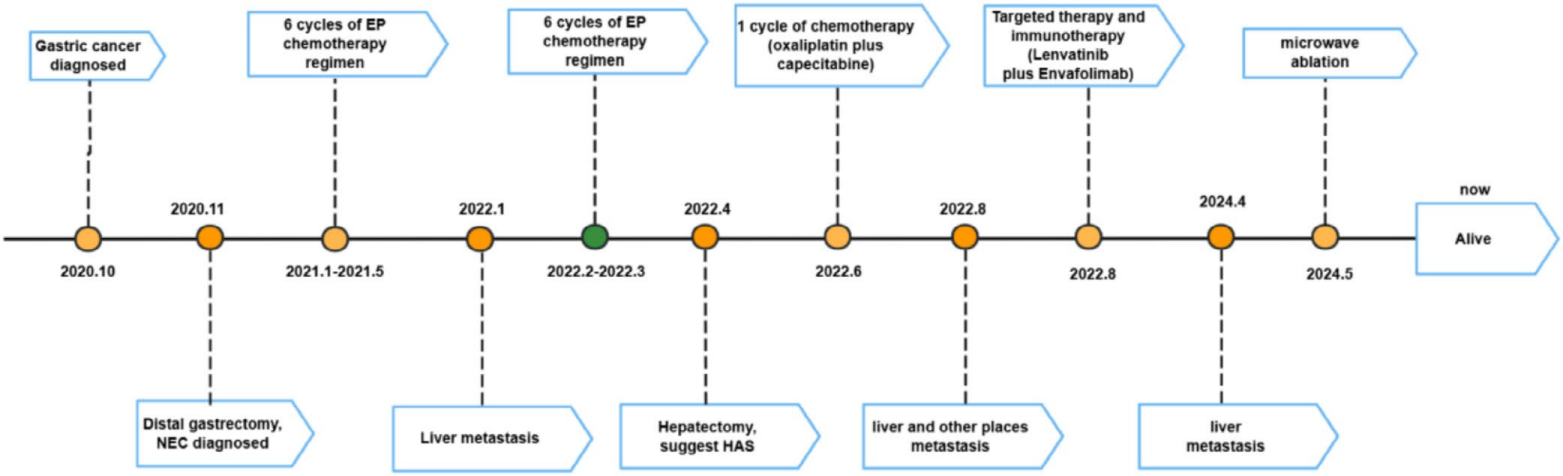
Timeline of the case report.

This therapeutic strategy has been implemented for 2 years and has demonstrated superior clinical efficacy in this patient, as evidenced by a significant inhibition of metastasis and focal lesion growth compared to previous treatments. In February 2023, the patient developed irritable bowel syndrome, but no other significant treatment‐related toxicities were observed during the treatment, resulting in high adherence to the ongoing chemotherapy regimen. Biochemical analyses conducted in 2024 indicated a slight decrease in blood cell count, hemoglobin, and lactic dehydrogenase levels, alongside a slight increase in urea and uric acid levels. MRI in April 2024 revealed an additional right posterior lobe inferior segmental nodule of the liver, while PET/CT shows a nodule in the right posterior lobe of the liver with increased metabolism, supposing a new metastasis (Figure 3). Immunization tests in May 2024 showed: Ckpan (AE1/AE3)(+), CK7(−), HepPar‐1(+), Arginase‐1(−), CD34(blood vessel+), Ki67 (+, 70%), SALL4(+), CDX‐2(+), CgA(+), Syn(+), RB(−), P53 (−), INSMI1(+). Next‐generation sequencing (NGS) gene detection showed: MYC copy number increased by 4, TP53(+), MSS. We treated him with microwave ablation of right hepatic space‐occupying lesions. From initial diagnosis to present, the patient has achieved a survival duration of approximately 5 years (Figure 4).

## 3 Conclusion and Results

There is a paucity of robust evidence regarding the indications and types of systemic chemotherapy, as well as the selection of drugs or drug combinations, for patients with advanced HAS and NEC [12]. In patients with NEC accompanied by liver metastases, local therapies such as arterial embolization or radiofrequency ablation have demonstrated a 50% response rate. In cases involving extrahepatic metastases, it has been shown that cytotoxic chemotherapy regimens, including streptomycin combined with 5‐fluorouracil or cyclophosphamide, doxorubicin alone or in combination with 5‐fluorouracil, dacarbazine, or temozolomide, oxaliplatin in combination with capecitabine, or 5‐fluorouracil in combination with calcium folinate, as well as molecularly targeted agents such as bevacizumab, sorafenib, sunitinib, and pazopanib, may be utilized as systemic therapy. These approaches may stabilize the disease or modestly extend progression‐free survival; however, there is no evidence indicating an improvement in overall survival [13]. In recent years, novel therapeutic options have emerged. In May 2024, W.E. Cruz Diaz et al. conducted a retrospective study demonstrating that the TEMCAP chemotherapy regimen (Capecitabine plus Temozolomide) appears to be a viable first‐line option for patients with advanced gastroenteropancreatic neuroendocrine tumors (GEP‐NET) [14]. Y. Matsubara et al. demonstrated that standard second‐line chemotherapy with ramucirumab after platinum‐containing chemotherapy may be effective for gastrointestinal NEC, especially G‐NEC [15]. Currently, there is no unified standard for the first‐line treatment of HAS. However, platinum‐based chemotherapy regimens (such as cisplatin combined with etoposide) are considered the standard first‐line treatment for metastatic HAS [16]. In recent years, neoadjuvant chemotherapy combined with immunotherapy has also shown promising clinical outcomes [17]. Here we exemplify some cases of NEC as well as HAS (Tables 1 and 2).

**TABLE 1:** Cases of NEC patients with different treatment regimens and their follow‐up results.

Year	Publication	Age (years)	Sex	Treatment	Follow‐up (months)	Outcome
2012 [20]	Clin Colorectal Canc	63	Male	Platinum‐based combination chemotherapy with carboplatin/paclitaxel as well as long‐acting release octretide	10	Dead
2014 [21]	Oncol Lett	65	Male	Laparoscopic‐assisted D2 radical total gastrectomy, Roux‐en‐Y esophagus‐jejunum anastomosis and FOLFOX chemotherapy	12	Alive
2017 [22]	Rev Esp Enferm Dig	57	Male	Ivor‐Lewis esophagectomy	8	Alive
2018 [23]	World J Gastroentero	74	Male	Irinotecan, CAV regimen and paclitaxel	31	Dead
2019 [24]	Medicine	60	Male	Radical gastrectomy and XELOX adjuvant chemotherapy	Unknown	Alive
2019 [25]	Medicine	38	Male	ESD	11	Alive
2021 [26]	World J Clin Cases	59	Male	Surgery, etoposide plus cisplatin	6	Alive
2024 [27]	Chin J Metastatic Cancer	65	Male	Surgery	14	Alive
2025 [28]	Chin J Clin Exp Patho	59	Female	Etoposide, Cisplatin	30	Alive

Abbreviations: CAV, cyclophosphamide, doxorubicin and vincristine; ESD, endoscopic submucosal dissection; FOLFOX, oxaliplatin, folinic acid calcium and fluorouracil; XELOX, oxaliplatin plus capecitabine.

**TABLE 2:** Cases of HAS patients with different treatment regimens and their follow‐up results.

Year	Publication	Age (years)	Sex	Treatment	Follow‐up (months)	Outcome
2017 [29]	BMC Cancer	61	Male	Combination chemotherapy with S‐1	8	Alive
2021 [30]	J Gastrointest Oncol	62	Male	Trastuzumab plus chemotherapy in the first line and paclitaxel plus ramucirumab in the second line	18	Unknown
2022 [31]	World J Clin Cases	72	Male	Pembrolizumab and bevacizumab	16	Alive
2022 [32]	Front Surg	66	Female	Oxaliplatin, gimeracil, and oteracil potassium capsules, paclitaxel, gimeracil, and oteracil potassium capsules, Sintilimab, paclitaxel, and regorafenib (oxaliplatin, folinic acid calcium salt hydrate, and 5‐FU)	16	Dead
2023 [33]	Front Surg	48	Female	Oxaliplatin plus S‐1 and PD‐1 inhibitor Terelizumab	14	Alive
2023 [34]	World J Clin Cases	61	Male	Docetaxel, oxaliplatin, capecitabine in the first line, and irinotecan, S‐1 plus apatinib in the second line	27	Dead
2023 [35]	Int J Surg Case Rep	70	Male	Capecitabine with oxaliplatin	72	Alive
2024 [36]	J Int Oncol	54	Female	Camrelizumab, Surufatinib	22	Alive

*Note:* S‐1 tegafur, gimeracil, and oteracil potassium.

In patients with gastrohepatoid adenocarcinoma, HER2 overexpression and TP53 mutations are frequently observed. Some studies have suggested that individuals with this condition may derive clinical benefit from anti‐ERBB2 or anti‐PD‐1 therapy. However, the patient in question, who was also diagnosed with neuroendocrine carcinoma, tested negative for HER2 expression and was therefore not considered for anti‐HER2 therapy. Initially, the patient underwent a chemotherapy regimen that included a cisplatin‐based drug following gastric cancer surgery. However, the treatment's efficacy was deemed suboptimal, as evidenced by the development of liver metastases. Consequently, the treatment was modified to a standard adjuvant therapy regimen post‐gastric cancer surgery, comprising oxaliplatin in combination with capecitabine. Notably, oxaliplatin is also indicated for metastatic hepatocellular carcinoma. Unfortunately, the patient experienced intolerance to capecitabine, necessitating a final adjustment to the treatment regimen, which involved the combination of immunotherapy and targeted therapy (Lenvatinib plus Envafolimab). In this revised therapeutic approach, Lenvatinib, recognized as a first‐line therapeutic option for liver cancer patients, was employed, demonstrating clinical efficacy comparable to that of sorafenib. The patient's second genome sequencing showed PD‐L1:CPS:3, with studies suggesting that the PD‐1/PD‐L1 pathway is a potential therapeutic target for gastric neuroendocrine cancer [18]. Therefore, we tried to combine Lenvatinib with Envafolimab, an immunosuppressant competing for PD‐L1, in order to achieve better disease control. Lenvatinib is a multi‐target tyrosine kinase inhibitor (TKI) that can effectively block tumor angiogenesis and reduce tumor blood supply, thereby inhibiting tumor growth. Envafolimab is a PD‐L1 inhibitor that restores the activation and proliferation of T cells and enhances the ability of T cells to recognize and kill tumor cells. In gastrointestinal malignancies, the synergistic interplay between anti‐angiogenic therapy and anti‐PD‐L1 immunotherapy has been increasingly recognized and validated by numerous researchers. Preclinical studies have demonstrated that anti‐angiogenic agents, such as Lenvatinib, can normalize tumor vasculature, enhance immune cell infiltration, and remodel the immunosuppressive tumor microenvironment, thereby sensitizing tumors to immune checkpoint inhibitors. Conversely, PD‐L1 blockade can further promote vascular normalization through interferon‐γ (IFN‐γ) and chemokine signaling, creating a positive feedback loop that amplifies antitumor immunity. Clinically, combination regimens involving anti‐angiogenic drugs and PD‐L1 inhibitors have shown promising efficacy in various digestive system tumors, including hepatocellular carcinoma, gastric cancer, and colorectal cancer, with improved objective response rates and prolonged survival. High expression of PD‐L1 is usually associated with a better response rate. Although PD‐L1 is not highly expressed in this patient (PD‐L1:CPS:3), some patients with low or negative PD‐L1 expression may still respond to or benefit from immunotherapy [19].

**FIGURE 5:**
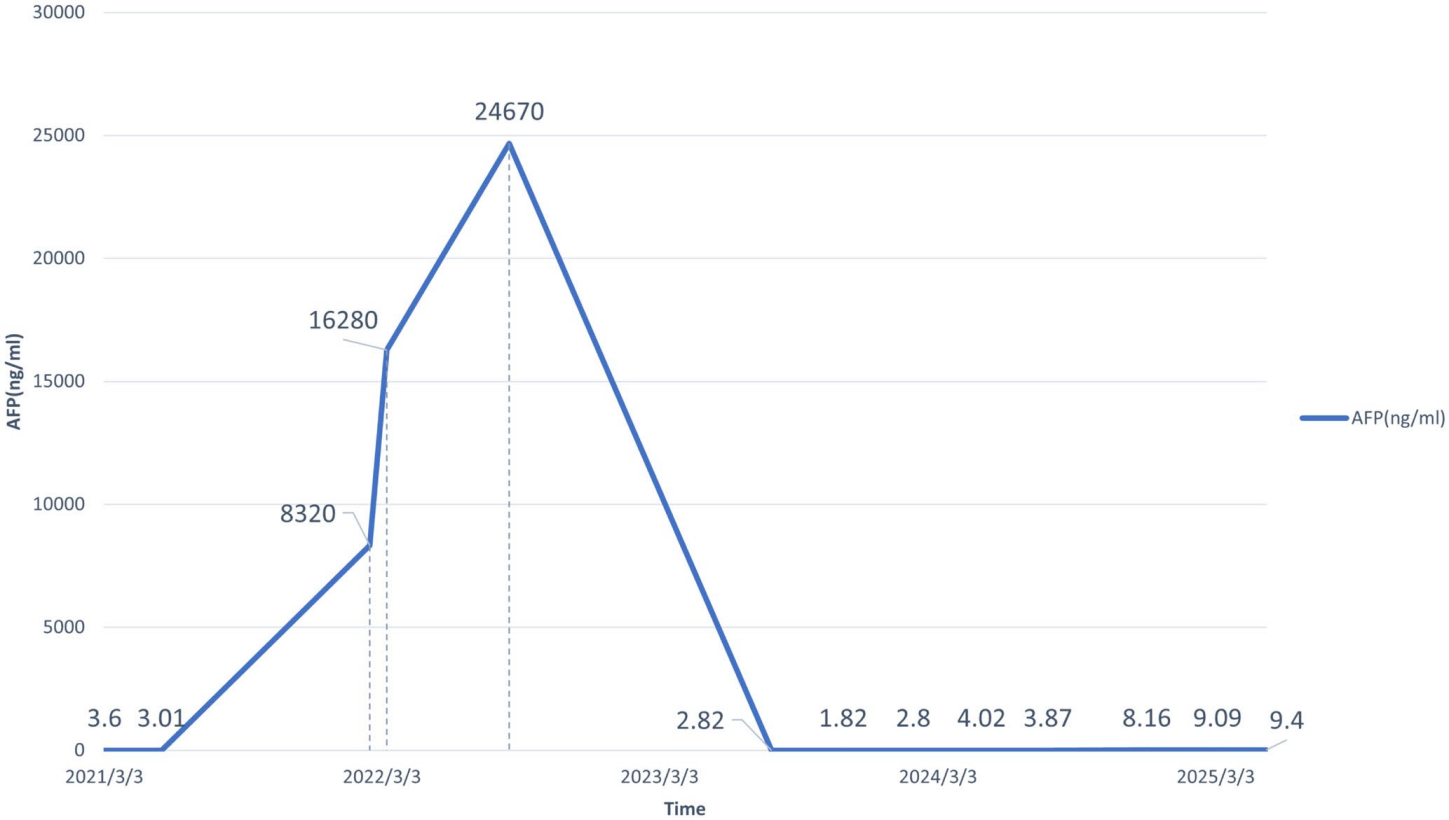
The dynamic changes in the patient's AFP levels over time.

The AFP levels of the patient in February, March, and August 2022 were recorded at 8320, 16,280, and 24,670 ng/mL, respectively. Following the initiation of targeted combined immunotherapy in August 2022, the patient's AFP levels progressively declined to within the normal range. The most recent AFP measurement in September 2024 was 4.44 ng/mL (Figure 5). Additionally, in May 2024, microwave ablation was performed on the right hepatic space‐occupying lesions. Prior to the ablation procedure, MRI conducted in April 2024 indicated that the diameter of the lesions in the lower segment of the right posterior lobe of the liver was approximately 1.0 cm. Post‐ablation, in July 2024, MRI revealed that its dimensions were approximately 3.2 × 2.5 cm, suggesting possible changes following the ablation treatment for liver metastasis. Upon re‐evaluation in September 2024, MRI demonstrated that the lesion had reduced to approximately 3.2 × 2.0 cm, which may be attributed to the effects of the ablation treatment. Since HAS and NEC are rare subtypes of gastric cancer, no identical case has been reported in the current literature to date. Our experience in diagnosis and treatment may offer instructive guidance for future cases with similar presentations.

## 4 Discussion

Surgery is traditionally considered the optimal treatment for localized diseases; however, chemotherapy frequently serves as the primary modality for managing highly aggressive malignant tumors characterized by poor prognoses and advanced‐stage presentation. Adjuvant chemotherapy or targeted combination immunotherapy may influence disease progression to some extent. In this case, the patient was diagnosed with hepatoid adenocarcinoma of the stomach concurrent with neuroendocrine carcinoma. Currently, there is a paucity of scientific treatment guidelines for these rare and highly invasive malignancies. The therapeutic regimen implemented in this case, comprising Lenvatinib and Envafolimab, may offer potential benefits for this condition. Since the initiation of this treatment protocol, the patient has not exhibited significant intolerance or adverse symptoms, and there are indications of disease stabilization. However, the emergence of new liver metastases underscores the necessity for further clinical and experimental investigations to comprehensively assess the efficacy of this treatment approach.

## Author Contributions

**Banghui Ma:** investigation, writing – original draft. **Ping Zheng:** resources. **Yongdong Jin:** conceptualization, project administration, resources, supervision, writing – review and editing.

## Consent

Informed written consent was obtained from the patient for publication of this report and any accompanying images.

## Conflicts of Interest

The authors declare no conflicts of interest.

## Data Availability

All raw data and code are available upon request.
